# Galectin-3: Integrator of Signaling via Hexosamine Flux

**DOI:** 10.3390/biom15071028

**Published:** 2025-07-16

**Authors:** Mana Mohan Mukherjee, Devin Biesbrock, John Allan Hanover

**Affiliations:** Cell Biochemistry Section, Laboratory of Cell and Molecular Biology, National Institute of Diabetes, Digestive and Kidney Diseases (NIDDK), National Institutes of Health, Bethesda, MD 20892, USA; devin.biesbrock@nih.gov

**Keywords:** HBP, UDP-GlcNAc, *N*-Glycan, *O*-GlcNAcylation, Galectin-3

## Abstract

Galectin-3 (Gal-3) is a β-galactoside-binding lectin that mediates diverse signaling events in multiple cell types, including immune cells. It is also a prognostic indicator for multiple clinically important disorders, including cardiovascular disease. Gal-3 binds to cell surface glycans to form lattices that modulate surface receptor signaling and internalization. However, the tissue-specific regulation of Gal-3 surface expression remains poorly understood. Here, we review evidence for the involvement of Gal-3 in cell surface signaling, intranuclear events, and intracellular trafficking. Our focus will be on the *O*-GlcNAc modification as a regulator of Gal-3 biosynthesis, non-canonical secretion, and recycling. We argue that the nutrient-driven cytoplasmic hexosamine biosynthetic pathway (HBP) and endomembrane transport mechanisms generate unique pools of nucleotide sugars. The differing levels of nucleotide sugars in the cytosol, endoplasmic reticulum (ER), and Golgi apparatus generate differential thresholds for the responsiveness of *O*-GlcNAc cycling, N- and O-linked glycan synthesis/branching, and glycolipid synthesis. By regulating Gal-3 synthesis and non-canonical secretion, *O*-GlcNAc cycling may serve as a nexus constraining Gal-3 cell surface expression and lattice formation. This homeostatic feedback mechanism would be critical under conditions where extensive glycan synthesis and branching in the endomembrane system and on the cell surface are maintained by elevated hexosamine synthesis. Thus, *O*-GlcNAc cycling and Gal-3 synergize to regulate Gal-3 secretion and influence cellular signaling. In humans, Gal-3 serves as an early-stage prognostic indicator for heart disease, kidney disease, viral infection, autoimmune disease, and neurodegenerative disorders. Since *O*-GlcNAc cycling has also been linked to these pathologic states, exploring the interconnections between *O*-GlcNAc cycling and Gal-3 expression and synthesis is likely to emerge as an exciting area of research.

## 1. Overview of Gal-3: A Unique β-Galactoside Binding Lectin

**Figure 1:**
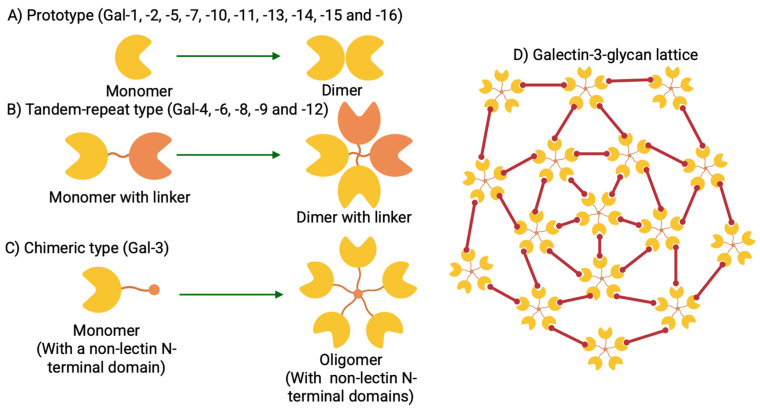
Schematic presentation of galectin family members and the galectin–glycan lattice. Galectin members are divided into three types based on the organization of the galectin carbohydrate recognition domain (CRD). (**A**) Prototype galectins (Gal-1, -2, -5, -7, -10, -11, -13, -14, -15, and -16). (**B**) Tandem repeat type galectins (Gal-4, -6, -8, -9, and -12). (**C**) The unique chimeric type of galectin (Gal-3). (**D**) Schematic presentation of bivalent lattice formation of the Gal-3 pentamer with glycans.

Lectins are classified into families, which include the galectins, an ancient and particularly interesting family. All members of the galectin family were numbered by order of discovery. Galectins are defined by evolutionarily conserved amino acid sequences and by their ability to recognize β-galactoside structures [1]. Galectins are omnipresent in sponges, fungi, nematodes, insects, and vertebrates, including mammals, and viral galectins have even been identified [1]. Their homologs are also present in plants, but not in yeast. To date, 16 mammalian galectins have been identified, each featuring a conserved carbohydrate recognition domain (CRD) comprised about 130 amino acids [2]. Galectins are classified into three subgroups based on the number and structural organization of their CRDs, in addition to their functional characteristics. The first subgroup includes prototype galectins made of non-covalently associated homodimers but which contain a single CRD (Gal-1, -2, -5, -7, -10, -11, -13, -14, -15, and -16) (Figure 1A) [2]. The second subgroup includes the tandem-repeat galectins (Gal-4, -6, -8, -9, and -12), and these heterodimers carry two distinct but homologous CRD motifs separated by a peptide linker sequence of up to 70 amino acids (Figure 1B) [2]. Lastly, the third subgroup comprises the tri-modular chimeras, containing a single canonical CRD connected to an unusually long, intrinsically disordered, N-terminal proline- and glycine-rich domain. This third subgroup is uniquely represented by Gal-3 (Figure 1C) [2]. Gal-3, initially named CBP35, is the only galectin discovered in vertebrates that can pentamerize (Figure 1C). It is also among the most intensively studied galectins [3]. It exerts distinct pleiotropic effects, contributing critically to a wide range of pathophysiological processes [4]. Galectins are soluble proteins with no transmembrane domains. Secreted galectins remain tethered to the cell surface through interactions with cell-surface glycoproteins or glycolipids. Remarkably, these secreted galectins, particularly the multivalent Gal-3, can mediate their functions by forming ordered interactions with multivalent glycan ligand structures, often termed as galectin lattices (Figure 1D) [5]. The formation of these lattices is thought to be a major contributor to cellular signaling [5,6].

## 2. Tissue Distribution of Gal-3 and Associations with Human Disease

**Figure 2:**
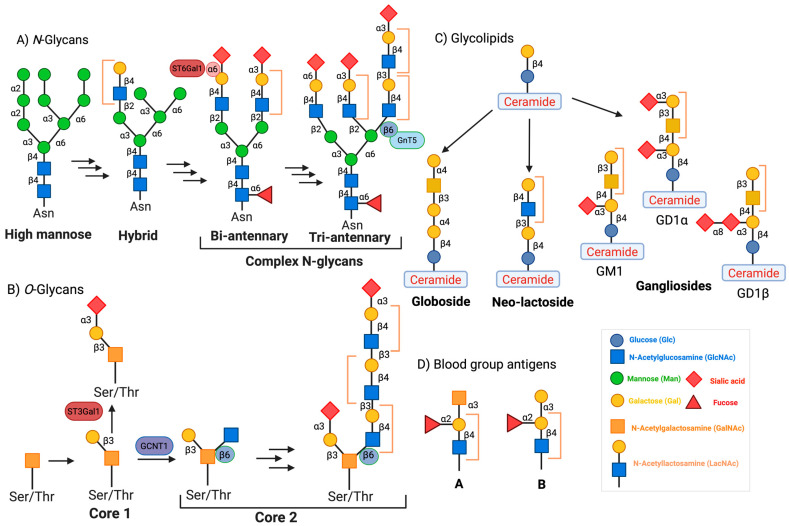
Schematic presentation of Gal-3 binding glycans. Gal-3 recognizes and binds to the galactose moiety of the Gal β1-4 GlcNAc linkage present in (**A**) *N*-glycans, (**B**) *O*-glycans, (**C**) glycolipids, or (**D**) blood group antigens. Brackets represent Gal-3 binding *N*-acetyllactosamine (LacNAc) sites on the glycan species. Asn = Asparagine, ST6Gal1 = α (2-6) sialyltransferase 1, GCNT1 = *N*-acetylglucosaminyltransferase 1, GnT5 = *N*-acetylglucosaminyltransferase 5, ST3Gal1 = α (2-3) sialyltransferase 1.

**Figure 3:**
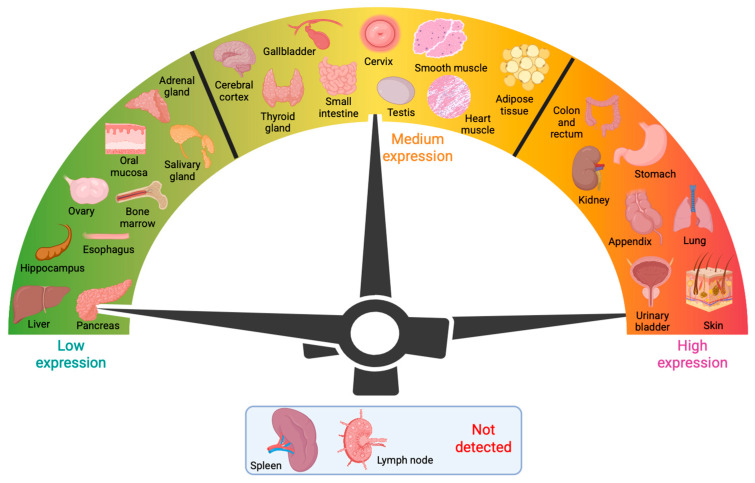
Schematic presentation of Gal-3 expression and distribution in mammalian tissues. Gal-3 is ubiquitously and differentially expressed throughout all tissues except the spleen and lymph nodes.

Gal-3 recognizes the distinct galactose β1-4 *N*-acetylglucosamine (*N*-acetyllactosamine) residues present in *N*- and *O*-glycans, glycolipids, or blood group antigens (Figure 2A–D). Gal-3 is encoded by the *LGALS3* gene, which is expressed in many different cell types, including small intestinal epithelial cells; corneal, conjunctival, and olfactory epithelia; colonic epithelia; epithelial cells of the kidney, lung, breast, thymus, and prostate; ductal cells of the salivary glands and pancreas; intrahepatic bile ducts; fibroblasts; chondrocytes; osteoclasts; osteoblasts; keratinocytes; Schwann cells; gastric mucosa; and endothelial cells from various tissues and organs. *LGALS3* is also expressed in cell types involved in the immune response, such as neutrophils, eosinophils, basophils, and mast cells; Langerhans cells; dendritic cells; and monocytes and macrophages from different tissues, but not in lymph nodes or spleen (Figure 3) [2,4]. During embryogenesis, Gal-3 expression is regulated in a tissue-specific and temporally dynamic manner. In the early stages of embryogenesis, Gal-3 expression is restricted primarily to the epithelial tissues, including the developing kidney, chondrocytes, and liver [2,7]. Intriguingly, Gal-3 knockout mice are viable, showing no apparent abnormalities, except for their premature senescence [8,9].

Although Gal-3 is predominantly located in the cytoplasm, it can shuttle between the cytoplasm and nucleus, a pathway involving import and export signals at the CRD and N-terminal tail (NT) of Gal-3 [10,11]. Ligands of Gal-3 are expressed ubiquitously throughout the cell and can be classified based on their localization [4]. Nuclear ligands include Gemin-2, -3, and -4; ALG-2 linked protein x (Alix) or ALG-2 interacting protein-1 (AIP-1); and survival of motor neuron protein. These ligands regulate gene transcription and promote pre-RNA splicing [7,12,13], but Gal-3 does not interact with RNA directly [14]. Other intracellular ligands for Gal-3 include cytokeratins, CBP70, Chrp, Gemin-4, Alix/AIP-I, and BCL-2. These ligands play a key role in cell survival. Their interaction with Gal-3 is a protein-protein interaction rather than a lectin–glycoconjugate interaction [12,13,15,16]. Extracellular Gal-3 ligands include laminin, fibronectin, tenascin, chondroitin sulfate, Mac-2 binding protein, and cell adhesion molecules like integrins. The Gal-3 ligands are engaged in modulation of cell–cell interactions, cell differentiation, inflammation, host defense, and many other cellular functions [7,17,18,19,20,21].

The multifunctional protein Gal-3 is expressed in many different tissues, engages in multiple biological events, and has been suggested to play a pivotal role in numerous diseases and clinical conditions. On average, over 500 scientific papers on Gal-3 are published yearly, and the numbers are constantly increasing [https://pubmed.ncbi.nlm.nih.gov/?term=galectin-3&filter=datesearch.y_1] (accessed on 20 May 2025). This fact alone, and that many articles in the most recent year discussed the role of Gal-3 in various pathophysiological conditions and diseases, attests to its growing potential relevance to human pathology. Gal-3 is a widely used prognostic indicator for many diseases, including asthma, atherosclerosis, atopic dermatitis, cerebral infarction, and chronic obstructive pulmonary disease. In addition, involvement of Gal-3 has been discovered in the following pathologies and disorders: aortic stenosis, endometriosis, enteric nervous system disorders, encephalitis, fibrosis, gastritis, HIV infection, inflammation, interstitial lung diseases, juvenile idiopathic arthritis, liver fibrosis, non-alcoholic fatty liver disease, obesity, pneumonia, pulmonary hypertension, plaque psoriasis, Q fever, rheumatoid arthritis, sepsis, systematic sclerosis, urinary tract infection, venous thrombosis, and especially heart failure, renal diseases, diabetes mellitus, and various cancers [4,7]. It must be emphasized that Gal-3 has not been causally linked to any of these disorders. However, it may be helpful as a measurable indicator of disease progression.

Expression of Gal-3 is disturbed in fibrosis, inflammation, and immunity, which has led to its extensive investigation for potential involvement in fibrotic diseases of the heart and kidney. Targeting Gal-3 is a possible therapeutic approach to slow the progression of these fibroses, which may advance into more severe heart and renal failure [7]. While not recommended on its own as a prognostic biomarker for individuals with heart failure, Gal-3 does show prognostic value in combination with other already established heart failure biomarkers [22]. Notably, elevated Gal-3 levels have been found in nearly all types of cardiovascular disease, not just heart failure. Elevated serum Gal-3 levels were associated with cardiovascular mortality and a greater incidence of cardiovascular events in patients with coronary heart disease [23,24]. Moreover, Gal-3 is also associated with new-onset atrial fibrillation, myocardial infarction (MI) size, and left ventricular remodeling in patients with a history of MI [25,26,27]. In addition to its role in cardiovascular disease, Gal-3 is also implicated in renal failure and associated complications [28]. Increased Gal-3 expression was detected in the serum and tumor tissues of renal cell carcinoma patients, as well as in patients with chronic kidney disease [29,30,31,32]. Furthermore, elevated levels of mRNA encoding Gal-3 were observed in a rat model of folic-acid induced acute renal failure [28].

## 3. Gal-3 Structure, Conservation, and Function

The N-terminal domain (NTD) of Gal-3 is composed of 110 to 130 amino acids depending on the species. This relatively flexible structure contains multiple repeats, each of which includes a consensus sequence, Pro–Gly–Ala–Tyr–Pro–Gly, followed by three additional amino acids [5]. The NTD of Gal-3 is highly conserved in different species. The initial sequence with 12 amino acids, preceding the Pro/Gly-rich repetitive domain, which is also called the small N-terminal domain, is particularly highly conserved in all mammalian Gal-3 forms. Human Gal-3 is characterized by the 21-amino acid-long NTD containing two Ser residues that can be phosphorylated, followed by nine non-triple-helical collagen-like Pro/Gly-rich repeats (I-IX), which harbor cleavage sites for diverse proteases [19]. The NTD and the nine repeats form the N-terminal tail. At least two functional characteristics are ascribed to the Gal-3 N-terminal tail: deletion of 11 amino acids following the first methionine block the secretion of Gal-3 [32]; mutation of the conserved Ser6 affects Gal-3-initiated anti-apoptotic signaling [33]. The NTD has also been implicated in the extracellular Gal-3 secretion [34]. In full-length Gal-3, the NTD is connected to the C-terminal CRD, composed of about 130 amino acids, forming a globular structure responsible for the lectin activity of Gal-3 [35,36]. It is evident from nuclear magnetic resonance experiments that these three sections, i.e., the NTD, collagen-like repeats, and the CRD, likely cooperate in a not yet clearly defined manner to account for their special role within the galectin network [37].

## 4. Gal-3 Nuclear Import and Export

Intracellular trafficking of Gal-3 is complex, and some reports of its cell biology are contradictory. Analysis by in vitro nuclear import assays using digitonin-permeabilized cells revealed that Gal-3 could be imported into the nucleus by either passive diffusion, *N*-terminal region-dependent active transport, or importin-α/β complex-dependent transport [11,33]. Studies using site-directed mutagenesis and truncation of full-length Gal-3 suggested that the CRD of Gal-3 is essential for its nuclear localization [10,34]. Transfections of the BT-549 human breast carcinoma cell line with serine-to-alanine (S6A) and serine-to-glutamic acid (S6E) mutants of Gal-3 revealed that phosphorylation of Ser172 in the NTD by casein kinase 1 favors Gal-3 export from the nucleus [35,36]. Secretion of Gal-3 to the extracellular space proceeds via a so-called non-classical pathway that is independent of the ER-Golgi complex and which involves both the NTD and segments of the collagen-like repeat region [36,37,38,39]. Gal-3 is transported to the early/recycling endosomes and is then partitioned into two routes: recycling back to the plasma membrane or targeting to the late endosomes/lysosomes. Systematic comparisons of various N- and C-terminally truncated forms of full-length Gal-3 lead to the conclusion that the CRD, NTD, and collagen repeating units are all required for the binding and endocytosis of Gal-3. Neither the CRD nor the N-terminal half of the protein, which comprises the NTD and the collagen-like internal repeating domain, alone can govern binding and endocytosis. Although the collagen-like domain is largely irrelevant to Gal-3 trafficking to the early/recycling endosomes, it is required for targeting Gal-3 to the late endosomes/lysosomes [40].

The biology of Gal-3, its application as an omnipotent diagnostic/prognostic tool, and the fact that it is the ‘alarm protein’ in the hierarchy of immune responses have already been well studied and reviewed [4,7,41,42,43,44,45,46,47,48,49]. In the review presented herein, we will focus on the regulation of the Gal-3 transcriptional and secretory mechanisms by hexosamine biosynthetic flux. We will also present the link between the latter and protein or lipid glycosylation.

## 5. Gal-3 Trafficking and Secretion

**Figure 4:**
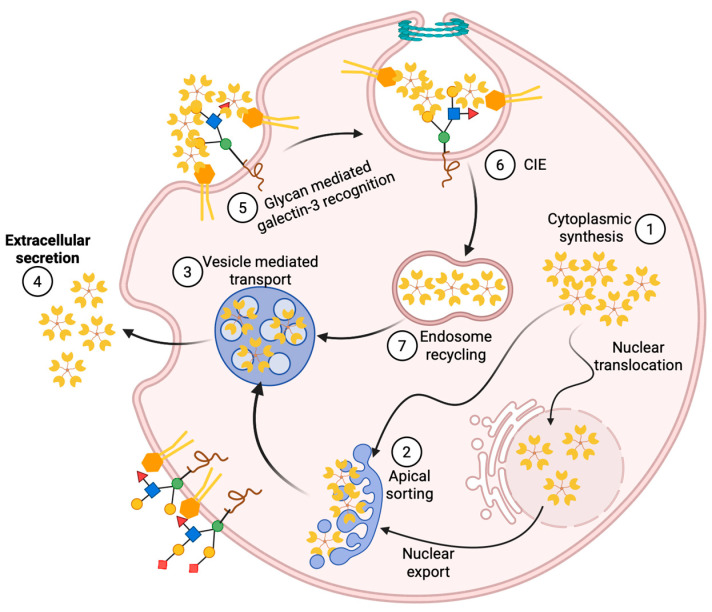
Schematic presentation of Gal-3 trafficking, showing its cytoplasmic synthesis, nuclear translocation, apical sorting, noncanonical secretion, glycan-mediated clathrin-independent endocytosis (CIE), and endosomal recycling. (**1**) Cytoplasmic synthesis of Gal-3 followed by nuclear import and export; (**2**) Apical sorting of Gal-3; (**3**) Gal-3 transports through exosomes as a form of noncanonical secretion; (**4**) Gal-3 is secreted into the extracellular matrix; (**5**) Glycan-mediated Gal-3 recognition occurs at the cell surface; (**6**) Gal-3 internalizes through CIE; (**7**) Internalized Gal-3 is recycled at the endosomes.

Mechanisms for the cellular export of galectins are not clearly understood. As we already mentioned, Gal-3 circumvents the classical “ER-Golgi apparatus” export route [39] and is secreted via a non-canonical mechanism (Figure 4). Research on the Gal-3 secretory mechanism has pointed to different routes for secretion, but it is possible that secretion can employ more than one pathway [50]. To date, four distinct non-conventional transport mechanisms have been suggested in order to explain how this non-conventionally secreted protein leaves the cell [51]. The first one is direct translocation of the protein across the plasma membrane to the extracellular milieu, as is best studied in the case of fibroblast growth factor 2. The second pathway for non-conventional protein secretion involves the sequestration of a soluble cytoplasmic factor by secretory lysosomes, as is shown for interleukin 1β. Vesicle-mediated secretion by microvesicles or exosomes covers the third suggested potential secretory pathway of Gal-3 [50]. Transmembrane proteins, which are inserted in the ER membrane but reach the plasma membrane without passing through the Golgi, are typically transported via the fourth type of pathway [52]. Findings from Mehul and Hughes, who described how an acylated Gal-3/chloramphenicol acyltransferase (CAT) chimera is released by microvesicle shedding in COS-7 cells, implied the third pathway’s implication in Gal-3 export [38]. Additionally, several proteomic studies identified Gal-3 in exosomes derived from different cell types [53,54,55,56]. Thus, it seems that Gal-3 may use distinct pathways for non-conventional secretion, and these may differ depending on cell type. However, the exact secretion mechanism of Gal-3 has not been identified for any of these pathways. Nevertheless, Hughes’ lab demonstrated how a short octapeptide in the N-terminal sequence of Gal-3 was necessary, but not sufficient, for the non-conventional secretion of an acylated fusion protein formed of CAT and the N-terminal residues (1–120) of hamster Gal-3 [39]. Many factors are known to affect Gal-3 secretion, e.g., heat shock, calcium ionophores, acylation, phosphorylation, and covalent modification by the attachment of O-GlcNAc [37,38,57,58]. Intracellularly, phosphorylation and importin-mediated mechanisms appear to be involved in nucleo-cytoplasmic shuttling of Gal-3 [35,59,60,61], while synexin-mediated mechanisms are indicated in Gal-3 translocation to the mitochondria [62]. It has been reported that deletion of the first twelve residues in the NTD of Gal-3 blocks its export into the cell supernatant [36]. Delacour et al. propose that it is not the NTD itself, but rather oligomerization, that is necessary for Gal-3 secretion, as the first twelve residues of the NTD in Gal-3 are responsible for the oligomerization of Gal-3 [63].

## 6. Gal-3 and Cell Surface Signaling via Lattice Formation

One of the important functions galectins play extracellularly is in the organizing of the cell membrane via forming macroscale interaction networks, often referred to as galectin lattices [4]. Gal-3 is especially important in organizing the galectin lattices, as the multivalence of its pentameric form enables the creation of large Gal-3 networks [3]. In solution, Gal-3 is monomeric unless high enough concentrations are reached, or bi- to multivalent ligands are present that serve as a core for aggregation [35,36,42,64,65,66,67,68,69,70,71,72]. The mode of Gal-3 functional oligomerization is a conundrum. Depending on which domain is involved, the interactions are categorized as type-N [64,65,73,74,75] or type-C [65,66,67,68] for the NTD- or CRD-mediated self-associations. Both the NT and the CRD play functional roles in this intermolecular assembly to varying extents [76,77,78,79]. Interactions between galectin and glycans play important roles in clathrin-independent endocytosis (CIE) [69,70]. Unlike well-characterized clathrin-mediated endocytosis, with its extensive intracellular machinery revealed [80], CIE is still poorly understood, i.e., little is known about the cytoplasmic components that regulate it [81,82,83,84]. CIE cargo comprises glycosylated proteins, like most membrane-bound proteins [85]. Attempts have been made to identify both intracellular regulators of CIE as well as plausible modulation of the galectin lattice on the extracellular side of the membrane via well-established galectin–glycan interactions. The galectin–glycan interactions can exert either a stimulatory or an inhibitory effect on CIE. The stimulatory role, as shown by Johannes’ group, is due to the initiation of direct interactions between Gal-3 and glycolipids, specifically glycosphingolipids (GSLs) bearing terminal sialic acid residues (gangliosides), thus inducing membrane bending. The bending was demonstrated both on protein-free giant unilamellar vesicles and in cells, during raft-dependent endocytosis or pit formation through clathrin-independent carriers [86,87]. The pentameric conformation of Gal-3, which is induced by high-density glycolipid ligands, might have geometric features that promote GSL clustering, membrane bending, and raft-dependent endocytosis [86]. Gal-3 promotes the formation of endocytic pits, resulting in stimulated internalization of the hyaluronan receptor (CD44) [86,87,88]. Gal-3 was also reported to stimulate the internalization of β-1 integrin by CIE [89]. In contrast, glycan-galectin interactions may impede internalization of epidermal growth factor receptor (EGFR) via galectin lattice–mediated cell-surface sequestration of the receptor, i.e., via the CIE [90,91,92,93,94,95]. CD59 and MHC-I, like CIE cargo, are sensitive to changes in galectin–glycan profiles, and interactions can be modulated by the metabolic flux and nutrient availability [96].

These two modes of action (Gal-3 promoting β-1 integrin internalization by endocytic pit formation and impeding internalization of EGFRs) act in opposing directions. There have been hints, however, that these two functions may not be mutually exclusive and could rather represent two ends of a spectrum [71], as illustrated in Figure 4. Gal-3-induced effects occur on a continuum from stimulatory to inhibitory, with distinct CIE cargo proteins exhibiting unique responses. In addition, regarding the spectrum of possible Gal-3 effects on cells, different cell types may stand at different positions on this spectrum [69]. At the apical membrane of polarized epithelial Madin–Darby canine kidney cells, Gal-3 was endocytosed to recycling endosomes, following an activation of a raft-independent pathway, in a lactose- and pH-dependent manner [72,76]. Further interactions between this internalized Gal-3 with glycosylated cargo in the apical recycling endosomes enable sorting of glycoproteins and transport them to the apical membrane [72,77]. Gal-3 export via exosomes has also been proposed [78], but a recent genome-wide CRISPR screening for Gal-3 secretory machinery demonstrated a free form of secreted Gal-3, rather than exosome-bound. Therefore, the exosomal route of Gal-3 export was not its primary secretory pathway [79].

A model termed the ‘desialylation glycoswitch’ has been introduced by Johannes and coworkers [80]. A reversible EGFR-controlled regulatory circuit based on glycans with terminal sialic acid (Sia) residues was proposed. The circuit would provide, through EGF-induced desialylation and Gal-3-dependent retrograde trafficking of plasma membrane glycoproteins, access to the Golgi apparatus to reset glycoprotein sialylation status. According to this model, EGF-induced CIE of Gal-3 occurs through the triggered removal of terminal Sia attached to *N*-glycans on cargo client proteins. The extracellular cues are relayed to galectin-driven endocytosis and transduced into pathophysiological responses through the acute remodeling of cell surface glycans. The same authors have established that EGF-induced Gal-3 binding to EGFR and tyrosine kinase activity of EGFR both directly depend on the presence of EGF glycan branches, i.e., on the removal of Sia by membrane-bound sialidases (neuraminidases). Following EGF-induced desialylation of EGFR glycan branches by the action of neuraminidases -1 and -3, which were stimulated by the acidification of the extracellular milieu via sodium-proton antiporter NHE1, integrins are transported via the retrograde route to the Golgi apparatus. Here, the glycan make-up of the integrins is reset by resialylation, and their function is repurposed by the polarized secretion of integrins to the leading edge of the invasive cell to support their migration. Gal-3 trafficking, therefore, may adopt a form of non-canonical secretion from the cytoplasm but also a glycan-dependent raft-enabled endocytosis to recycling endosomes, where Gal-3 plays a significant role in post-Golgi sorting.

## 7. Hexosamine Biosynthetic Pathway (HBP) and Gal-3

**Figure 5:**
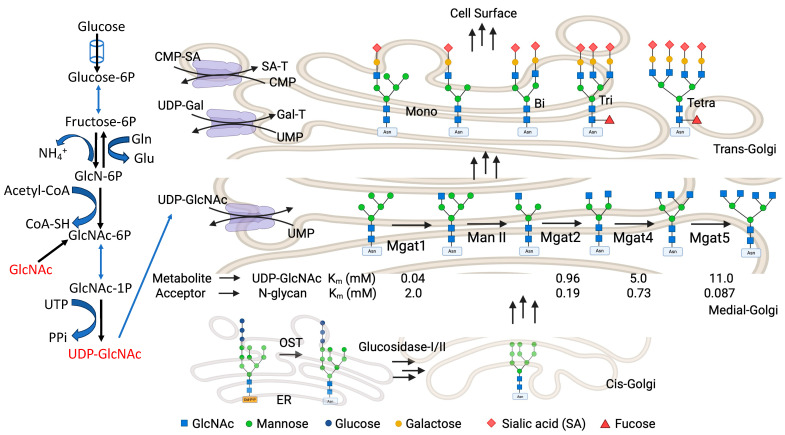
The HBP generates the nucleotide sugar donor UDP-GlcNAc. Oligosaccharyltransferase (OST) transfers the preassembled donor Glc_3_Man_9_GlcNAc_2_-pp-dolichol to NXS/T motifs on nascently synthesized proteins in the ER. Glycoproteins travel from the ER to the cis, medial, and trans Golgi *en route* to the cell surface. The Golgi-residing *N*-acetylglucosaminyltransferases, named after the names of their encoding genes (Mgat1, Mgat2, Mgat4, and Mgat5), generate branched *N*-glycans that display a range of affinities for galectins. The Michaelis constant (Km, a measure of enzyme affinity for its substrate) values for Mgat1, Mgat2, Mgat4, and Mgat5 are indicated for both UDP-GlcNAc and acceptor glycoproteins as measured in vitro. Gln = Glutamine; Glu = Glutamate; GlcN-6P = Glucosamine-6-phosphate; GlcNAc = N-Acetyl-D-glucosamine; UTP = Uridine triphosphate; PPi = Pyrophosphate; UDP = Uridine diphosphate; UMP = Uridine monophosphate; CMP = Cytidine monophosphate; Man II = Golgi mannosidase II; Mgat1, 2, 4, and 5 = N-acetylglucosaminyltransferases I, II, IV, and V.

The HBP is a branch of the main glycolytic pathway, starting with glucose (Glc) and ending with uridine-diphosphate-N-acetylglucosamine, UDP-GlcNAc (Figure 5). It is an essential pathway of amino sugar synthesis. The HBP integrates the key metabolic pathways of glucose, amino acids, nucleosides, and lipids, thereby serving as a nutrient rheostat. Cells employ HBP flux to monitor nutrient availability in physiological and pathological conditions, even though only 2–5% of the glucose taken up by cultured cells end up in the HBP. As mentioned, the end-product of the HBP is UDP-GlcNAc, a high-energy nucleotide sugar donor, which serves as a substrate for the biosynthesis of both O-Glycans and N-glycans of glycoproteins, glycolipids, proteoglycans, and glycosaminoglycans. UDP-GlcNAc cellular dynamics are strictly regulated by mechanisms involved in multistep enzymatic reactions. Not only is the HBP an essential pathway for UDP-GlcNAc synthesis, but also an essential conduit that regulates the biosynthesis of other nucleotide sugars. The end-product of the HBP, UDP-GlcNAc, can be further epimerized by UDP-galactose-4-epimerase (GALE) to generate UDP-GalNAc, which is used in biosynthesis of glycans that will be attached to mucin-type O-glycoproteins, complex glycolipids, and proteoglycans [81]. Additionally, UDP-GlcNAc is metabolized by UDP-*N*-acetylglucosamine 2-epimerase/*N*-acetylmannosamine kinase (GNE) to produce *N*-acetylmannosamine (ManNAc), and furthermore Sia, manose (Man), and fucose (Fuc). The nucleotide sugars: CMP-Sia, GDP-Man, and GDP-Fuc, are essential donors for biosynthesis of N-glycans (Figure 5), glycolipids, proteoglycans, and glycosaminoglycans. The enzymatic steps involved in biosynthesis of nucleotide sugars are depicted in Figure 5. An aberrant flux of glucose or GlcNAc results in an altered UDP-GlcNAc concentration through the HBP, leading to a change in glycoprotein conformation, oligomerization, trafficking, localization, turnover, and stability of the glycan-Gal-3 lattice.

## 8. *N*-Glycan Branching and Gal-3

Gal-3 is evolutionarily conserved and is ubiquitously expressed with a distinct binding partner, the Galβ1-4GlcNAc (or LacNAc) moiety [1]. LacNAc is a recurring disaccharide in branched *N*-glycans, which represent the major Gal-3 ligands at the cell surface [6,82,83]. In vertebrates, ∼30% of the transcriptome is translated in the secretory pathway. On the luminal side of the ER membrane, most de novo synthesized proteins are *N*-glycosylated. In mammalian glycoproteins, over 97% of *N*-glycans are covalently attached to the Asn residue within the conservative motif Asn-X-Ser/Thr (where X can be any amino acid but Pro) [84]. The initial *N*-glycan is transferred from the glycolipid Glc_3_Man_9_GlcNAc_2_-pyrophosphate-dolichol to the Asn residue of the nascent protein by the action of an enzyme system known as the oligosaccharyltransferase complex (OST) [85]. The Glc_3_Man_9_GlcNAc_2_ *N*-glycan is a ligand for ER chaperone proteins, including calnexin and calreticulin, which promote protein folding efficiency, and aid in protein quality control and secretion or degradation of misfolded proteins [86,87]. After the initial folding in the lumen of the ER, where the three Glc residues are consecutively trimmed by respective glucosidases, most glycoproteins transition to the Golgi apparatus. There, mannosidases I and II consecutively trim the Man residues, thus forming the Man_5_GlcNAc_2_–Asn core structure [85,88]. The latter is further remodeled by *N*-acetylglucosaminyltransferases, which form branches in *N*-glycans in an ordered enzymatic cascade comprising enzymes called Mgat1, Mgat2, Mgat4a/b/c, and Mgat5 [89]. Each of them transfers one GlcNAc unit in a specific linkage to *N*-glycans, which is followed by the consecutive addition of a β-linked galactose. Therefore, each branch is a potential ligand for galectin since it ends with LacNAc. Although the affinity of galectins for *N*-glycans increases with the extent of branching and abundance of poly-LacNAc extensions (repeating Galβ1-4GlcNAcβ-units) [90], the lattice formation is a complex process rather than a simple combination of binding affinities. For example, a single glycoprotein with three *N*-glycans and nine LacNAc moieties shows a range of Gal-3 binding constants within a range encompassing 1000-fold [91]. In addition, Gal-3 oligomeric lattices formed through its NTD have been visualized on the cell surface, and they varied depending on the cell state [75].

Galectins bind to omnipresent glycans attached to cell surface glycoconjugates; hence, they may appear to lack specificity for individual ligands. Indeed, proteomic analyses of Gal-3-interacting partners (N-cadherin and glycosphingolipids), Phytohemagglutinin-L (LPHA)-binding proteins (the higher affinity galectin ligands), and Mgat5-dependent raft association indicated that Gal-3 is able to bind many glycoproteins simultaneously [71,92,93]. Lau et al. studied how the extent of *N*-glycosylation and the degree of branching depend on the flux of UDP-GlcNAc. Moreover, they investigated Gal-3 affinity for differentially branched *N*-glycans and how branching can regulate cell proliferation and differentiation [70]. Their systematic data obtained both in silico and in vitro fit into a model linking the evolutionary origins of the multiplicity of *N*-glycans attached to growth factor receptors (GFRs) and the adjustment of metabolic flux through the HBP, which regulates *N*-glycan branching in the Golgi apparatus. They showed that variations in the hexosamine flux through exogenous addition of GlcNAc, especially at hyperphysiological concentrations, affect the residence time of the half-life of receptors on the cell surface by modulating the interactions of branched *N*-glycans with the Gal-3 lattice. The relative affinity of the Gal-3 lattice for *N*-glycans bound to transmembrane glycoproteins is proportional to the number of *N*-glycosylation sites (Asn-X-Ser/Thr) and is sensitive to the modification through the Golgi *N*-glycan branching pathway. The avidity of Gal-3 for *N*-glycosylated proteins increases with the number of attached *N*-glycans. Glycoforms produced in the Golgi complex are above and below the affinity threshold for stable association with the Gal-3 lattice. Being ultrasensitive to hexosamine flux, the Golgi system attaches primarily tri- and tetra-antennary *N*-glycans to glycoproteins with a high number of *N*-glycosylation consensus sites. Given the high avidity of Gal-3 lattices for these multi-branched *N*-glycans, their complexes restrict *N*-glycoprotein endocytosis, hence hindering the downregulation of signaling [82].

With a change in UDP-GlcNAc concentration, there is a subtle change in *N*-glycan branching. The enzymes involved in *N*-glycan branching show an intriguing pattern in the recognition of their substrates. The branching enzymes (Mgat1 to Mgat5) show a different binding affinity for their common substrate UDP-GlcNAc (∼300-fold). The Michaelis constant (Km) changes from 0.04 to 11 mM for Mgat1 and Mgat5, respectively. Mgat1 has a low affinity for the acceptor *N*-glycan at ~2 mM, and this relationship is reversed for Mgat4 and Mgat5 (Figure 5). With Mgat1 functioning near saturation and Mgat4 and Mgat5 operating well below their Km, this makes the synthesis of tri- and tetra-antennary glycans highly sensitive to the supply of UDP-GlcNAc [82]. The Dennis group performed computational modeling and found that the sequential decreasing efficiency of Golgi enzyme reactions and the removal of intermediate products from the Golgi are the two major conditions required for *N*-glycan branching ultra sensitivity. However, all the experiments were conducted with hyper-physiological concentrations of GlcNAc, and hence UDP-GlcNAc, which is much higher than that required to form the glucosyl donor for *O*-GlcNAc. It was estimated that, if the ER and Golgi concentrate UDP-GlcNAc from 10- to 30-fold, the concentration of the UDP-GlcNAc in the cytoplasm, nucleus, and mitochondria is in the range of 2–30 μM, near the known Km for *O*-GlcNAc transferase (*OGT*) and the UDP-GlcNAc transporters [94]. Thus, overexpression of UDP-GlcNAc transporter should lead the responses to occur at lower concentrations of exogenous GlcNAc, and this remains a subject for future investigations. Additionally, high UDP-GlcNAc flux will also result in hyper *O*-GlcNAcylation [95,96], altered GSL synthesis [97], and augmented mucin-type glycan formation [98], which in turn can alter the Gal-3 lattice. Furthermore, glucose, glutamine, and acetyl-CoA supply the hexosamine pathway, regulating the biosynthesis of UDP-GlcNAc and *N*-glycan branching, and thereby regulating glycoprotein retention in the galectin lattice [99,100].

## 9. *O*-GlcNAc Regulation of Gal-3

*O*-glycans covalently bound to proteins are also structurally diverse. They can be further subclassified based on the sugar attached, including *O*-GalNAcylation, *O*-GlcNAcylation, *O*-fucosylation, *O*-mannosylation, and O-glucosylation [101,102]. Among these glycosylations, the two most abundant types of *O*-glycosylations are the structurally diverse mucin-type *O*-glycosylation (attachment of *O*-GalNAc to Ser or Thr) occurring in the Golgi apparatus and *O*-GlcNAcylation, the installation of a single sugar residue, *O*-GlcNAc, which occurs in the nucleus, cytoplasm, and mitochondria [103,104,105].

*O*-GlcNAcylation is an abundant post-translational modification (PTM) in mammals [104]. More than 15,000 proteins from 43 different species have been identified with *O*-GlcNAc modifications [106]. *O*-GlcNAcylation modulates protein synthesis, subcellular localization, stability, and protein–protein interactions, thereby influencing diverse cellular processes at the molecular level. Interplay between O-GlcNAcylation and other PTMs markedly amplifies the complexity of protein regulatory networks [105,107]. O-GlcNAcylation occurring at Ser/Thr serves to adjust the extent of Ser/Thr phosphorylation in proteins [108,109,110].

A defining characteristic of O-GlcNAc modification, setting it apart from most other PTMs, is its regulation by a single pair of antagonistic enzymes. OGT and *O*-GlcNAcase (OGA) are responsible for installation and removal of *O*-GlcNAc from proteins [111,112]. *O*-GlcNAcylation also appears to serve as a nutrient-sensing mechanism in cells. Elevated cellular glucose levels enhance glucose flux through the HBP. This pathway generates the precursor molecule UDP-GlcNAc, the nucleotide sugar donor substrate for *O*-GlcNAcylation. Consequently, elevated glucose concentrations lead to a boost in the supply of UDP-GlcNAc availability, thereby promoting increased protein *O*-GlcNAcylation [104]. The interplay between *O*-GlcNAcylation and phosphorylation, its dynamic regulation by just two enzymes, and its sensitivity to nutrient availability have collectively sparked significant interest in its roles in cellular signaling and diverse disease states. The nutrient-sensing function of *O*-GlcNAcylation is especially critical, as it allows cells to detect nutrient abundance and swiftly modulate cellular processes in response to changing metabolic conditions in order to rapidly adapt to fluctuations in nutrient availability [110,113]. This regulatory axis links metabolism to signaling, contributing to the fine-tuning of cellular functions and implicating *O*-GlcNAcylation in a broad spectrum of physiological and pathological contexts. Beyond its role in nutrient sensing, O-GlcNAcylation is involved in a broad range of biological processes, including gene transcription, epigenetic regulation, signal transduction, stress response, metabolic homeostasis, and immune function response [94,105,111,114].

Gal-3 is a nucleocytoplasmic protein that is also secreted into the extracellular space. Since Gal-3 is synthesized in the cytoplasm and translocates into the nucleus, it is exposed to the enzymes of *O*-GlcNAc cycling. Once reaching the surface, Gal-3 binds to the terminal galactose residue of the LacNAc (Gal-β-1,4-GlcNAc) substrate. An emerging question is whether the pathways of *O*-GlcNAc cycling may be linked by Gal-3 secretion. *O*-GlcNAcylation is known to change the functional activities of proteins and govern the trafficking and localization of proteins in cells [107,113]. *O*-GlcNAcylation occurs directly on key subunits of the protein complexes involved in generating COPII, COPI, and clathrin-coated vesicles (CCVs) [107]. Furthermore, some proteins in autophagy and non-conventional secretory pathways are reported to be regulated by *O*-GlcNAcylation [115,116]. There is growing evidence on the potential involvement of individual galectins in processes of cellular differentiation, but more work is needed to explore formal links to relevant *O*-GlcNAc cellular homeostasis [117].

## 10. Established Links Between O-GlcNAcylation and Gal-3 Activity

Alteration of the *O*-GlcNAcylation cycle with the OGA inhibitor Thiamet G (TMG) or the OGT inhibitor Ac-5SGlcNAc (AC) changed RNA expression of *LGALS3* in the adherent malignant breast cancer cell line MCF7 but not in the suspended acute promyelocytic leukemia cell line HL-60 [118]. Further investigation with HL-60 cells showed that neutrophilic differentiation of those cells induced by all-trans retinoic acid (ATRA) and 6-diazo-5-oxo-L-norleucine (DON, GFPT inhibitor) was associated with a significant drop in cellular *O*-GlcNAc levels in serum-contained and serum-free cell culture media. The amount of secreted Gal-3 (measured by ELISA assay) was significantly upregulated by ATRA and DON, while intracellular Gal-3 protein expression was upregulated by both ATRA and DON in serum-contained media and by all four treatments (ATRA, DON, TMG, and AC) in FBS-containing media (measured by RT-qPCR followed by Tukey’s multiple comparison analysis). A much higher magnitude of Gal-3 protein expression was observed in ATRA-induced serum-free cell culture media [119]. As a result of reduced O-GlcNAcylation, extraembryonic endoderm (XEN) cells promote galectin secretion and cell differentiation, whereas high *O*-GlcNAcylation in embryonic stem (ES) cells promotes intracellular accumulation of galectins [120]. Immunoassays (Western blot and ELISA) revealed that the XEN cells had lower concentrations of both intracellular and extracellular Gal-3 compared to the ES cells, and the relative secretion of Gal-3 was significantly increased as a result of *O*-GlcNAcylation occurring concomitantly with XEN differentiation. Label-free LC-MS/MS quantitative proteomic analysis of control and iOGA groups (*O*-GlcNAcylation dynamics disrupted with OGA inhibitor Thiamet G) identified altered Gal-3 expression in glioblastoma (GBM) secretome, predicting Gal-3 could be a substrate for *O*-GlcNAc addition and removal [121].

An interesting study with yellow-rumped warblers (*Setophaga coronata*) showed RNA expression of *LGALS3* significantly increased in flight muscles and hearts for the migratory flown (MF) cohort compared to the migratory unflown (MU) and winter unflown (WU) cohorts, while protein expression of the *O*-GlcNAcylated protein pool (detected with RL2 antibody) in flight muscle was higher in the case of WU compared to MF and MU [122].

**Figure 6:**
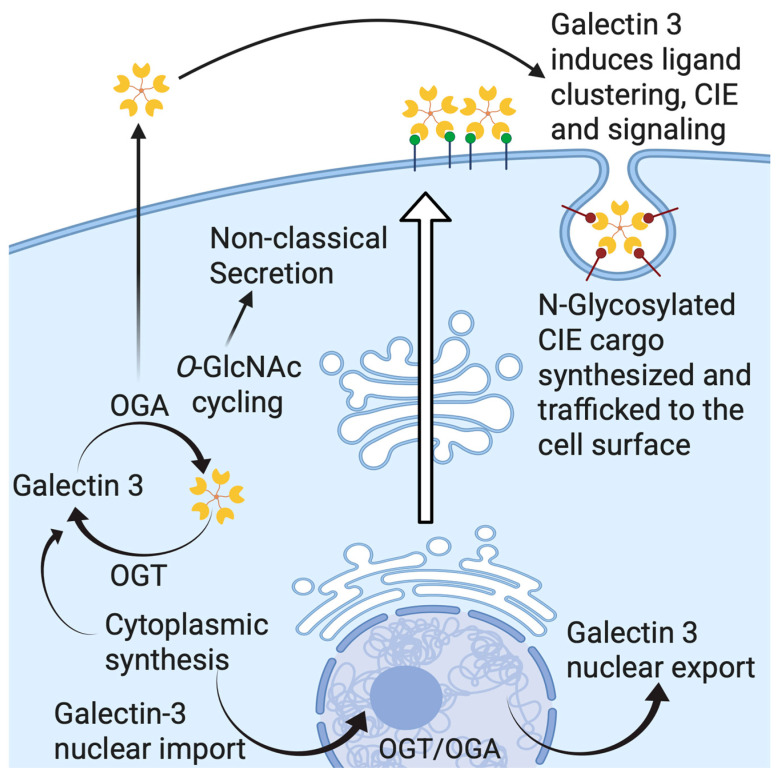
Schematic presentation of the role of *O*-GlcNAcylation in Gal-3 secretion. Gal-3 is synthesized in the cytoplasm and then secreted by noncanonical mechanisms. The cytoplasmic origin and nuclear translocation allow interaction with the *O*-GlcNAcylation machinery. This model indicates that Gal-3 necessitates *O*-GlcNAcylation and deglycosylation for proper secretion.

Using OGA WT and OGA KO mouse embryonic fibroblast (MEF) cells, our group has shown that Gal-3 indeed is a substrate for *O*-GlcNAc transferase, and its secretion is tightly regulated by *O*-GlcNAcylation dynamics and *O*-GlcNAc levels [58]. Immunoprecipitation of GFP-tagged Gal-3 and Western blotting have shown a significant difference in *O*-GlcNAcylation status between cytoplasmic and secreted Gal-3. A dramatic alteration in Gal-3 secretion was also observed in response to nutrient conditions, which were dependent on dynamic *O*-GlcNAcylation. Although no *O*-GlcNAcylation site is predicted for Gal-3 itself [123], the YinOYang server predicted six possible *O*-GlcNAcylation sites with a high degree of confidence. Five of these predicted sites are clustered in the multimerization region of Gal-3, and all the mutants that had all five of these sites deleted or mutated were secreted at a significantly lower level than control Gal-3 GFP. The last one (Ser 243) is adjacent to the nuclear export region, and mutation of this site did not appear to alter Gal-3 secretion. *O*-GlcNAcylation of Gal-3 may play a role in its secretion, and the removal of *O*-GlcNAc is an important step for regulation of Gal-3 multimerization and secretion. *O*-GlcNAc-driven alterations in Gal-3 secretion also facilitated changes in CIE. This indicates that dynamic *O*-GlcNAcylation of Gal-3 plays a role in modulating its secretion and can tune its function of transducing nutrient-sensing information coded in cell surface glycosylation into biological effects (Figure 6). Whether the effect of the *O*-GlcNAcylation cycle in the regulation of Gal-3 is only secretory, transcriptional, translational, or some combination is yet to be determined.

## 11. Discussion

Gal-3 is a widely studied protein with diverse binding targets mediating a wide variety of biological outputs. Gal-3 is ubiquitously expressed in various cell types and tissues and is detectable inside and outside of cells, as well as on the cell surface. Gal-3 is implicated in cell and cell–extracellular matrix adhesion, cell growth and differentiation, the cell cycle, cell signaling, apoptosis, and angiogenesis. Consequently, Gal-3 is involved in the regulation of development, immune reactions, tumorigenesis, and tumor growth and metastasis. Important questions remain concerning how regulation of Gal-3 expression may occur in different cell types under different physiological and pathophysiological conditions. The biological roles of Gal-3 have been initially attributed to its carbohydrate-binding activity, but during the past decade a whole new spectrum of functions unrelated to lectin activity has been revealed. Molecular mechanisms that drive the trafficking of Gal-3 in cells, as well as its secretion, remain elusive. This includes mechanisms of non-conventional secretion, since Gal-3 lacks the typical ER-signal peptide required for the conventional pathway.

**Figure 7:**
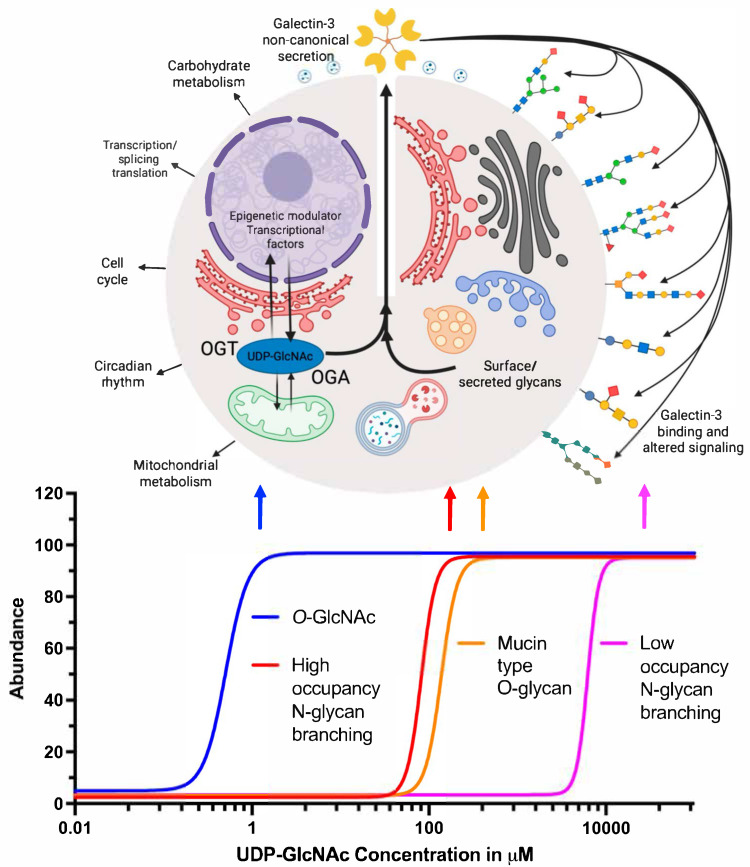
Gal-3 provides synergy between nutrient-driven *O*-GlcNAcylation and surface glycan signaling. At reduced intracellular concentrations of UDP-GlcNAc, dysregulation of the O-GlcNAcylation cycle may occur, leading to changes in Gal-3 transcriptional control and its subsequent secretion. These alterations can manifest across the physiologically relevant range of cytoplasmic concentrations, which are tightly regulated via feedback inhibition of the HBP. Due to the activity of specific nucleotide sugar transporters, UDP-GlcNAc concentrations within the ER and Golgi apparatus are substantially elevated, as evidenced by the higher substrate thresholds required for extensive *N*-glycan branching and mucin-type *O*-glycan biosynthesis. At elevated UDP-GlcNAc concentrations, low-affinity *N*-glycosylation sites are modified, leading to the synthesis of low-occupancy *N*-glycans. The threshold-dependent changes illustrated suggest that as *N*-glycan synthesis and branching increase, Gal-3 expression and secretion are restrained to prevent excessive activation of Gal-3–mediated signaling pathways.

The evidence presented here suggests that Gal-3 secretion could serve as a nexus linking nutrient information to cell surface signaling events. Our model reflects recent findings demonstrating that *O*-GlcNAc may regulate the expression and secretion of many factors, including Gal-3 (Figure 7). The model is predicated on the nucleotide sugar requirements for diverse surface glycan structures. In particular, the model treats UDP-GlcNAc concentrations in different cell organelles as a key variable. We also point out the importance of the Km values in glycan processing enzymes that utilize UDP-GlcNAc. Changes in UDP-GlcNAc levels linked to nutrient and stress conditions can occur in many cell types, such as activated T-cells. In our model, we argue for threshold effects in response to these changes. At lower levels of UDP-GlcNAc, perturbation of the *O*-GlcNAcylation cycle may occur, triggering altered Gal-3 transcriptional regulation and secretion. These changes can occur over the range of cytoplasmic concentrations that occur physiologically, and the levels are regulated by feedback inhibition of hexosamine synthesis. Owing to the presence of transporters, the ER and Golgi concentrations of UDP-GlcNAc are much higher, as reflected in the thresholds shown for high occupancy *N*-glycan branching and mucin-type *O*-glycan synthesis. Finally, at very high levels of UDP-GlcNAc, low occupancy *N*-glycans are synthesized. The threshold-driven changes we depict would suggest that as synthesis and branching of *N*-glycans occurs, Gal-3 synthesis and secretion would be limited to avoid hyper-activation of signaling pathways normally triggered by Gal-3. In this model, *O*-GlcNAc would serve as a homeostatic regulator of Gal-3-stimulated signaling. It should be noted that experimental thresholds for the synthesis of the various glycan forms have not been rigorously established. There is evidence for increased branching of glycans at high levels of hexosamine flux [82]. There is also evidence that this leads to an increase in Gal-3 binding and lattice formation. However, this model emphasizes that the different glycan species likely have different thresholds for assembling Gal-3 binding sites. The feedback mechanism illustrated is one means of self-limiting the signaling in response to Gal-3.

## 12. Conclusions

Although Gal-3 has been implicated in several debilitating disorders, its detailed mechanism of action has yet to be elucidated. To clarify the precise mechanisms of Gal-3 action, it is of the utmost importance that its physiologically functional ligands are identified. Moreover, the development of potent, low-molecular-weight inhibitors of Gal-3 could provide powerful analytical tools for investigating functions of Gal-3 [124,125]. These Gal-3 inhibitors, with high oral bioavailability and low toxicity profiles, could lead to combatting progressive tissue fibrosis and Gal-3-related heart failure. Alternatively, the O-GlcNAcylation regulation of Gal-3 could be targeted for altering Gal-3 secretion. Current understanding of the various binding partners of Gal-3 is also incomplete. Further characterization and visualization of the Gal-3 lattice utilizing advanced (optical) techniques is necessary to understand the exact mechanisms by which this regulatory protein influences various (extra)cellular processes.

## Data Availability

No new data were created or analyzed in this study. Data sharing is not applicable to this article.

## Abbreviations

HBP	Hexosamine biosynthetic pathway
UDP	Uridine diphosphate
GlcNAc	N-Acetyl-D-glucosamine
Gal-3	Galectin-3
CIE	Clathrin-independent endocytosis
CRD	Carbohydrate recognition domain
NTD	N-terminal domain
ER	Endoplasmic reticulum
